# Chasing environmental sustainability in healthcare organizations: insights from the Italian experience

**DOI:** 10.1186/s12913-025-13158-x

**Published:** 2025-07-26

**Authors:** Michela Bobini, Americo Cicchetti

**Affiliations:** 1 CeRGAS, Health and Social Care Management Research Center, SDA Bocconi School of Management, Milan, Italy; 2 Graduate School of Health Economics and Management, ALTEMS, Catholic University of the Sacred Heart, Rome, Italy; 3 Department of Economic and Business Management Sciences, Catholic University of the Sacred Heart, Rome, Italy

**Keywords:** Environmental sustainability, Healthcare organizations, Managerial commitment, Barriers and drivers, Healthcare management

## Abstract

**Background:**

Environmental sustainability is an increasingly critical priority for healthcare systems due to their substantial environmental impact and vulnerability to climate-related challenges. Yet, its integration within healthcare organizations remains inconsistent and poorly documented. This study investigates the level of awareness, the degree of implementation, and the extent of operationalization of environmental sustainability activities in healthcare organizations. It also identifies the main barriers and drivers influencing this process.

**Methods:**

A semi-structured survey was developed based on Rogers’ Diffusion of Innovations framework and the NHS England Green Plan model. It was administered to Italian healthcare organizations affiliated with the Italian Federation of Healthcare and Hospital Organizations. The survey was complemented by a desk analysis of strategic documents. The lack of explicit national policy on environmental sustainability in healthcare offered a neutral context to examine organizational approaches, motivations, and challenges in the absence of centralized guidance.

**Results:**

The survey achieved a 23% response rate, with 39 organizations from 14 regions participating. A high level of awareness among healthcare leadership was observed, with 97% of respondents reporting that environmental sustainability was recognized as a strategic concern. However, only 41% of organizations had formalized sustainability commitments in strategic documents. Despite this, 90% reported having undertaken at least one concrete action, indicating that operational engagement often precedes formal strategic planning. The analysis also revealed a positive association between the number of sustainability domains addressed and the number of actions implemented, suggesting that broader engagement supports more structured implementation.

**Conclusions:**

The findings suggest that organizations do not necessarily follow a linear or formalized pathway in adopting environmental sustainability practices. Instead, implementation often emerges from pragmatic and context-driven processes, even in the absence of strategic codification. This behavior reflects both the adaptability and the limitations of decentralized action, with risks of fragmentation and uneven progress. A national strategy—supported by standardized indicators, reporting systems, and financial incentives—could strengthen alignment, enhance scalability, and support healthcare organizations in embedding sustainability more systematically, while preserving flexibility for local innovation.

**Supplementary Information:**

The online version contains supplementary material available at 10.1186/s12913-025-13158-x.

## Introduction

Incorporating environmental sustainability (ES) in the healthcare sector is critical due to its significant impacts on both public health and the global environment. Romanello et al. [1] highlight that climate change intensifies health risks, such as heat-related illnesses and the spread of infectious diseases, which increasingly strain healthcare systems that are already destabilized by crises like COVID-19 and global conflicts. These compounded pressures underscore the need for climate-adaptive healthcare practices that reduce emissions and enhance resilience. The healthcare sector’s substantial environmental impact—contributing around 5.2% of global emissions—creates a reciprocal effect where the sector not only affects but is also vulnerable to climate-induced health crises [2]. A sustainable approach to healthcare, as Howard et al. [3] suggest, includes implementing low-carbon solutions that improve public health outcomes—such as cleaner air—and mitigate future climate-related health impacts. These actions are crucial as climate change exacerbates resource scarcities, disrupting supply chains essential to healthcare delivery. Embracing sustainable practices not only reduces the sector’s carbon footprint but also strengthens healthcare infrastructure to endure and respond to climate challenges, positioning healthcare as both a responder to and mitigator of climate-related health risks [1, 3–5]​​. Hospitals occupy a critical position in advancing ES within the healthcare sector, primarily due to their substantial consumption of resources, energy, and financial assets, compounded by their continuous 24/7 operations [6–14]. In addition to managing these intensive demands, hospitals are tasked with delivering safe, high-quality care in a cost-effective manner to ensure optimal health outcomes [15]. Consequently, ES practices in this context must be designed to integrate seamlessly with service quality objectives [12, 16]. This interdependence presents a distinct challenge, as hospitals are required to simultaneously balance service excellence, economic efficiency, and environmental responsibility, rather than addressing these dimensions in isolation [17–19]. ES practices in healthcare organizations target critical areas, including facility design, energy and water conservation, waste management, supplier selection, and promoting environmentally conscious behavior among staff [10–14, 17, 20–22]. The drive for sustainability appears to be driven by a diverse set of factors, including cost reduction, regulatory compliance, and a growing commitment to social responsibility [23, 24]. These elements collectively motivate healthcare leaders to integrate ES practices into organizational strategies and operations. In alignment with other sectors [25], healthcare organizations face considerable challenges, including operational inertia, resource constraints, a lack of standardized procedures, and insufficient training [10, 26, 27]. These factors collectively impede progress, limiting efforts to move beyond basic compliance with environmental sustainability standards. Addressing these barriers requires fostering a sustainability culture aligned with healthcare’s mission, ensuring both budget adherence and clinical quality [26].

While ES in healthcare has garnered increasing scholarly attention, substantial gaps persist in understanding how healthcare organizations adopt, implement, and operationalize ES activities in a structured and systematic manner. McGain et al. [12], through a systematic review, highlighted that existing research predominantly concentrates on technical aspects while overlooking critical dimensions related to organizational behavior, managerial engagement, and the integration of sustainability within governance and decision-making frameworks. Other studies [24, 28] have addressed sustainability from a broader perspective, encompassing environmental, social, and economic dimensions. These contributions reveal fragmented efforts, a progressive decline in strategic planning, and the absence of institutionalized frameworks to ensure the long-term integration of sustainability. Collectively, these studies [12, 23, 24, 28–31] point to a critical need for empirical research that moves beyond isolated cases or conceptual discussions, and instead investigates the current level of awareness, the degree of implementation, and the barriers and drivers influencing the integration of ES activities across healthcare organizations.

This study adopts an implementation science perspective [29], drawing on Rogers’ Diffusion of Innovations framework [32, 33], to investigate how healthcare organizations approach environmental ES. The primary objective is to understand the level of awareness, the degree of implementation, and the extent of operationalization of ES activities within healthcare settings. Specifically, the study aims to: (i) assess healthcare organizations’ awareness and engagement regarding ES; (ii) evaluate the current implementation status and integration of ES activities into organizational operations; (iii) identify the barriers and drivers in implementing ES initiatives. By addressing these objectives, this research provides insights into the strategic, managerial, and operational factors influencing the adoption and diffusion of ES activities within the healthcare sector, contributing to the broader discourse on how complex organizations navigate sustainability transitions.

## Methodology

### Study design

This study adopted a mixed-methods approach to assess the integration of environmental sustainability strategies within healthcare organizations. To achieve the stated objectives, a semi-structured survey was developed and administered to healthcare organizations. The survey design was informed by a comprehensive literature review, which identified key dimensions and questions relevant to the study [10, 12, 22, 23, 29]. The survey was developed according to specific requirements of questionnaire formatting [34–36] and was specifically created for this study, having not been previously published. It included 18 questions—both multiple-choice and open-ended (see Appendix)—and was administered through the Qualtrics.com platform. Estimated completion time was approximately 30 min, though it was shorter for organizations not engaged with environmental sustainability. Initially, the survey collected general information to establish a baseline understanding of the respondents’ demographic and organizational context. Participants were asked to provide details such as the name and location of their organization, as well as their professional role. Subsequently, the survey included questions regarding the presence of regional guidelines or policies that promote environmental sustainability in healthcare. This element was incorporated to assess the broader contextual framework of the organizations, as existing literature identifies such policies as critical drivers of sustainability initiatives [12, 23, 37–39]. The first section focused on assessing healthcare organizations’ engagement regarding ES and was designed around implementation science theories [32, 33, 40], with a particular emphasis on Rogers’ Diffusion of Innovations theory [32]. This framework describes the sequential stages an organization undergoes when adopting an innovation—in this case, the integration of ES activities. According to Rogers’ Diffusion of Innovations model, the stages have been reinterpreted to align with the dynamics of ES adoption within healthcare organizations. Specifically:

- **Persuasion**: The organization forms an opinion and develops a favorable attitude toward environmental sustainability. In this study, this stage corresponds to the recognition by top management of the relevance of ES within organizational management and strategic priorities.
- **Decision**: The organization commits to environmental sustainability. In the context of public healthcare organizations, where formal processes and documentation are essential, the commitment has been proxied by the formal inclusion of ES initiatives within official strategic documents (e.g., Integrated Plans of Activities and Organization - PIAO, Corporate Acts).
- **Implementation**: The organization translates its commitment into concrete actions. This stage reflects the operationalization of ES through the initiation of concrete activities and projects aimed at advancing environmental goals.

While initially developed to explain knowledge transfer in clinical practice, this theory has been increasingly applied to understand evidence adoption in healthcare policy and organizational settings [41–43]. Utilizing Rogers’ framework in this context provides a systematic approach to identifying the factors influencing the adoption of ES. Additionally, this section explored governance models adopted by organizations for ES, such as delegating responsibilities to existing personnel or teams, appointing dedicated sustainability or energy managers, or creating specialized teams. It also examined the professional expertise of individuals involved in sustainability efforts, such as backgrounds in economics, engineering, law, medicine, pharmacy, or other fields. The second section examined the extent to which healthcare organizations implement ES activities. Questions in this section were informed by NHS England’s Green Plan model [44], which is widely recognized as an international best practice. The survey assessed the implementation of ES across key domains defined by the Green Plan, including: Workforce and System Leadership, Sustainable Models of Care, Digital Transformation, Travel and Transport, Estates and Facilities, Medicines, Supply Chain and Procurement, Food and Nutrition, and Climate Adaptation. Furthermore, the section investigated specific actions undertaken by organizations, such as identifying objectives and priorities, developing monitoring systems, implementing SMART (specific, measurable, achievable, relevant, and time-bound) actions aimed at reducing environmental impacts, and engaging both internal and external stakeholders. The final section focused on identifying the barriers and enablers in implementing ES activities. Respondents were asked to reflect on challenges such as limited data availability, competing organizational priorities, absence of political mandates, or insufficient perceived value of sustainability initiatives among stakeholders. Using a Likert scale (1 to 10), participants evaluated the significance of these barriers. Open-ended responses were encouraged to provide nuanced insights into the organizational dynamics influencing the adoption of sustainability initiatives. In addition to the survey, the study included a desk analysis of strategic documents provided by the participating organizations, as well as national and regional environmental sustainability policies, to further contextualize the findings.

### Setting

The Italian National Health Service (SSN), established in 1978, was founded on principles of universal healthcare, human dignity, health needs, and solidarity, with healthcare primarily financed through general taxation [45]. This system provides nearly comprehensive healthcare coverage, though some services require user contributions. However, inefficiencies in central planning and escalating healthcare costs prompted significant reforms in the 1990 s, leading to the decentralization of authority to regional governments and the managerialization of the healthcare system. These reforms resulted in the creation of Local Health Authorities (LHAs), which are territorially based, and semi-independent Hospital Trusts, both accountable to regional governments. Additionally, the system includes public and private IRCCS (Scientific Institutes for Research, Hospitalization, and Healthcare), specialized institutions that focus on advanced care and research and receive targeted funding from the Ministry of Health. Despite its strengths, the SSN faces considerable challenges, including healthcare workforce shortages and aging infrastructure, compounded by demographic shifts such as an aging population and the rise of chronic diseases. These pressures were starkly highlighted during the COVID-19 pandemic, which exposed significant regional disparities, particularly in southern Italy, where bed shortages and limited community-based care services were more pronounced. In response, the Italian government, supported by the European Union’s National Recovery and Resilience Plan (PNRR), has initiated substantial investments to strengthen local healthcare services and accelerate the digitalization of the health system.

Italian environmental legislation operates within a broad framework, primarily shaped by the integration of international, EU directives, and agreements. Italy’s initiatives for environmental sustainability include strategic and legislative measures promoting a holistic ecological transition. The National Sustainable Development Strategy (SNSvS) serves as an action framework to harmonize economic, social, and environmental development, aligning national objectives with the UN’s SDGs. By integrating Agenda 2030 principles, SNSvS provides a roadmap for addressing urgent challenges like climate change, social inequalities, and the promotion of a circular economy. Italy is updating its Nationally Determined Contributions in line with the Paris Agreement, aiming to reduce greenhouse gas emissions by 55% by 2030 compared to 1990 levels, aligning with the EU Green Deal. The National Climate Adaptation Plan (PNACC) offers a framework to mitigate climate risks, improve socio-economic and natural resilience, and leverage new climate conditions. The Integrated National Energy and Climate Plan (PNIEC) establishes 2030 targets for energy efficiency, renewable sources, and CO₂ reduction, detailing the measures needed to meet these goals. The Strategy for the Energy Refurbishment of Buildings (STREPIN) outlines Italy’s real estate profile, identifying current and future energy renovation targets. In energy policy implementation, the role of the Energy Manager (Law n°10/1991, Art. 19) is crucial in both public and private sectors. The Public Administration’s Green Procurement Plan mandates the use of Minimum Environmental Criteria (CAM) in procurement processes, aiming to identify environmentally sound solutions. However, at the time of this study, no national policies explicitly addressed environmental sustainability in healthcare. This policy gap provided a neutral context for exploring how healthcare organizations conceptualized and implemented environmental sustainability strategies, offering valuable insights into their approaches, motivations, and challenges in the absence of centralized directives.

### Participants and data collection

Organizations were selected based on their membership to Italian Federation of Healthcare and Hospital Organizations (FIASO - Federazione Italiana Aziende Sanitarie e Ospedaliere) as of July 2024. FIASO is the leading representative association for healthcare and hospital organizations in Italy. The survey was distributed via email through the FIASO newsletter in July 2024. Along with the invitation to participate in the survey, participants were provided with a PDF version of the questionnaire, enabling them to collect the necessary information and engage relevant personnel to ensure comprehensive responses. To increase the survey’s representativeness, it was additionally distributed via email in September 2024 through the authors’ networks within the Veneto Region, where FIASO’s presence is more limited, as well as to IRCCS Policlinico Gemelli. The survey was received by 171 healthcare organizations (see Appendix). The link to the survey remained active from July 2024 to October 2024.

### Data analysis

The analysis of the data collected in this study employed both quantitative and qualitative approaches to provide a comprehensive understanding of the level of awareness, the degree of implementation, and the extent of operationalization of ES activities within healthcare settings. For the quantitative analysis, descriptive statistics were used to summarize key metrics, including the number of ES areas addressed, the frequency of actions implemented, and the distribution of engagement levels across organizations. For the qualitative analysis, responses to open-ended survey questions were examined using thematic analysis to identify recurring drivers and barriers influencing ES adoption. Additionally, strategic documents provided by the organizations were systematically reviewed to identify sustainability priorities, evaluate their alignment with operational practices, and highlight variations in the specificity and comprehensiveness of the reported strategies. By combining these analytical approaches, the study was able to provide a robust evaluation of how healthcare organizations are progressing toward ES and identify areas where further improvements are needed.

## Results

### Respondents

The survey was completed by 39 respondents from 14 regions (see Appendix): Abruzzo (*n* = 2), Campania (*n* = 2), Emilia-Romagna (*n* = 4), Friuli Venezia Giulia (*n* = 2), Lazio (*n* = 3), Liguria (*n* = 1), Lombardy (*n* = 10), Marche (*n* = 1), PA Bolzano (*n* = 1), Piedmont (*n* = 1), Sardinia (*n* = 1), Sicily (*n* = 2), Tuscany (*n* = 1), and Veneto (*n* = 9). The overall response rate was 23%, with significant variation across regions: while some areas, such as Veneto and the Autonomous Province of Bolzano, achieved full participation (100%), regions like Puglia, Calabria, and Basilicata did not register any responses (0%). Despite this seemingly modest percentage, it is consistent with response rates typically observed in similar survey-based studies [23, 24].

**Table 1:** Distribution of participant healthcare organizations in Italy by geographic area and organizational type

Area/Type	Local Health Authorities	Hospital Trust and IRCCS	Total
Northwest Italy	8 (21%)	4 (10%)	**12 (31%)**
Northeast Italy	10 (26%)	6 (15%)	**16 (41%)**
Central Italy	3 (8%)	2 (5%)	**5 (13%)**
Southern Italy and Islands	3 (8%)	3 (8%)	**6 (15%)**
Total	**24 (62%)**	**15 (38%)**	**39 (100%)**

The percentages are calculated relative to the total of 39 respondents, highlighting the regional and organizational diversity in the sample. For analytical purposes, Italian regions were grouped into four geographic areas: Northwest Italy, Northeast Italy, Central Italy, and Southern Italy and Islands, following the standard ISTAT classification.

The sample, as illustrated in Table 1, includes healthcare organizations from all major geographic areas of Italy—Northwest, Northeast, Central Italy, South, and Islands—ensuring that the findings capture the diversity of healthcare settings across the country. This includes regions with varying levels of infrastructure, resources, and organizational autonomy. The notable overrepresentation of Northern regions (Northwest and Northeast, accounting for 72% of the sample) reflects the actual concentration of healthcare infrastructure and resources in these areas. This aligns with well-documented geographic disparities within the Italian healthcare system, where Northern regions typically exhibit more advanced and systematically implemented sustainability practices. In terms of organizational diversity, the sample is well-structured, with Local Health Authorities comprising 62% of respondents, consistent with their central role in delivering healthcare at the regional level. The inclusion of Hospital Trusts and IRCCS (38% collectively) further enriches the analysis by incorporating the perspectives of research-focused and tertiary care institutions, ensuring a broader representation of organizational priorities and capacities.

The respondents primarily hold management roles or are professionals specifically appointed by organizational leadership due to their responsibilities or particular interest in environmental sustainability. A significant share (16 respondents) is involved in strategic governance, including positions such as general, medical, or administrative directors. Furthermore, a considerable number of participants occupy technical and operational positions, particularly within units dedicated to infrastructure management, clinical engineering (10 respondents), and organizational or technological innovation (6 respondents). The remaining respondents are engaged in specialized departments focusing on areas such as prevention and environmental safety.

### The progression of healthcare organizations in embedding environmental sustainability.

Table 2 maps the progression of healthcare organizations in embedding environmental sustainability (ES), following the adapted stages of Rogers’ innovation-decision model. 97% (38 out 39) of respondents reported that top management had acknowledged the relevance of ES in organizational management, suggesting high levels of awareness and positive attitudes toward sustainability within leadership teams. However, only sixteen (41%) organizations had formalized their commitment to ES in strategic documents, reflecting a gap between awareness and formal strategic integration. Despite this, thirty-five (90%) respondents indicated that their organizations had already taken concrete actions related to ES, demonstrating that most organizations are actively engaged in sustainability practices even without formalized strategic commitments.

**Table 2:** Summary of the different stages in the Innovation-Decision process for integrating ES among healthcare organizations

Stage of innovation decision process	Survey’s questions	Answers	
		Yes	No
*Persuasion*	Has top management recognized the relevance of environmental sustainability in organizational management?	38 (97%)	1 (3%)
*Decision*	Has the commitment to environmental sustainability been formalized in strategic documents?	16 (41%)	23 (59%)
*Adoption*	Has your organization taken any actions related to environmental sustainability?	35 (90%)	4 (10%)

### The establishment and dissemination of environmental sustainability objectives

Of the 16 participants who reported having formally expressed their intention to work on environmental sustainability, only 12 submitted the required documentation. The analysis of the 12 strategic documents provided by healthcare organizations highlights a clear distinction between general governance documents and dedicated sustainability-focused documents, as detailed in Table 3.

**Table 3:** Summary of strategic documents that include environmental sustainability

Document Type	Number of Documents
Integrated Plan of Activities and Organization (PIAO)	8
Corporate Act	1
Sustainability Report	2
Project Document Focused on Sustainability	1
**Total**	**12**

The majority (9 documents) included references to ES within institutional frameworks such as the Integrated Plan of Activities and Organization (PIAO) or the Corporate Act. Both are mandatory governance tools for Italian public healthcare entities, designed to define organizational structure, strategic objectives, and compliance with transparency and performance regulations. In these cases, sustainability is typically addressed as part of broader institutional principles, resulting in high-level commitments without detailed operational strategies. By contrast, a smaller number of organizations produced dedicated sustainability documents, including two Sustainability Reports and one project-specific document explicitly focused on environmental objectives. These documents generally present more concrete initiatives, measurable actions, and long-term planning, reflecting a more proactive approach to sustainability beyond formal compliance. Across all documents, recurring themes emerged, particularly in areas such as energy efficiency, waste management, digitalization, structural modernization, sustainable mobility, and green procurement. These priorities indicate a shared awareness of key environmental challenges within the healthcare sector. However, despite these common thematic areas, the depth and operationalization of sustainability strategies varied considerably. While a few organizations demonstrated structured approaches supported by performance indicators, many limited their engagement to general statements without clear implementation plans. This analysis suggests that, although environmental sustainability is increasingly acknowledged at a strategic level, only a limited number of healthcare organizations have developed specialized documentation that enables a deeper and more operational integration of sustainability practices.

### Governance and competence in environmental sustainability

The data reveal that 28 of the 35 organizations actively pursuing environmental sustainability efforts have implemented organizational measures. Among these, 12 organizations have recruited specialized technical personnel, emphasizing the growing recognition of the importance of expertise in addressing sustainability challenges. Additionally, 6 organizations have established multidisciplinary teams, reflecting a collaborative approach necessary for tackling the multifaceted nature of sustainability. In other cases, organizations have pragmatically assigned sustainability responsibilities to existing staff, relying on internal resources to meet environmental goals. While this approach demonstrates adaptability, it also underscores potential resource constraints, which could limit the long-term effectiveness of sustainability initiatives. The professional backgrounds of individuals involved in sustainability efforts reveal a pronounced emphasis on technical expertise. Engineering professionals, numbering 33, represent the majority, signaling a focus on technical and infrastructural solutions such as energy efficiency, waste management, and system optimization. Beyond engineering, representation from Medicine (11 professionals), Economics and Management (11 professionals), and Pharmacy (4 professionals) highlights efforts to embed sustainability into clinical operations and organizational management. Additional contributions from fields such as Law (3 professionals), Chemistry (1 professional), and Prevention Technicians enrich the governance framework, supporting compliance, chemical waste management, and risk mitigation.

### The implementation of ES in healthcare organizations

**Fig. 1:**
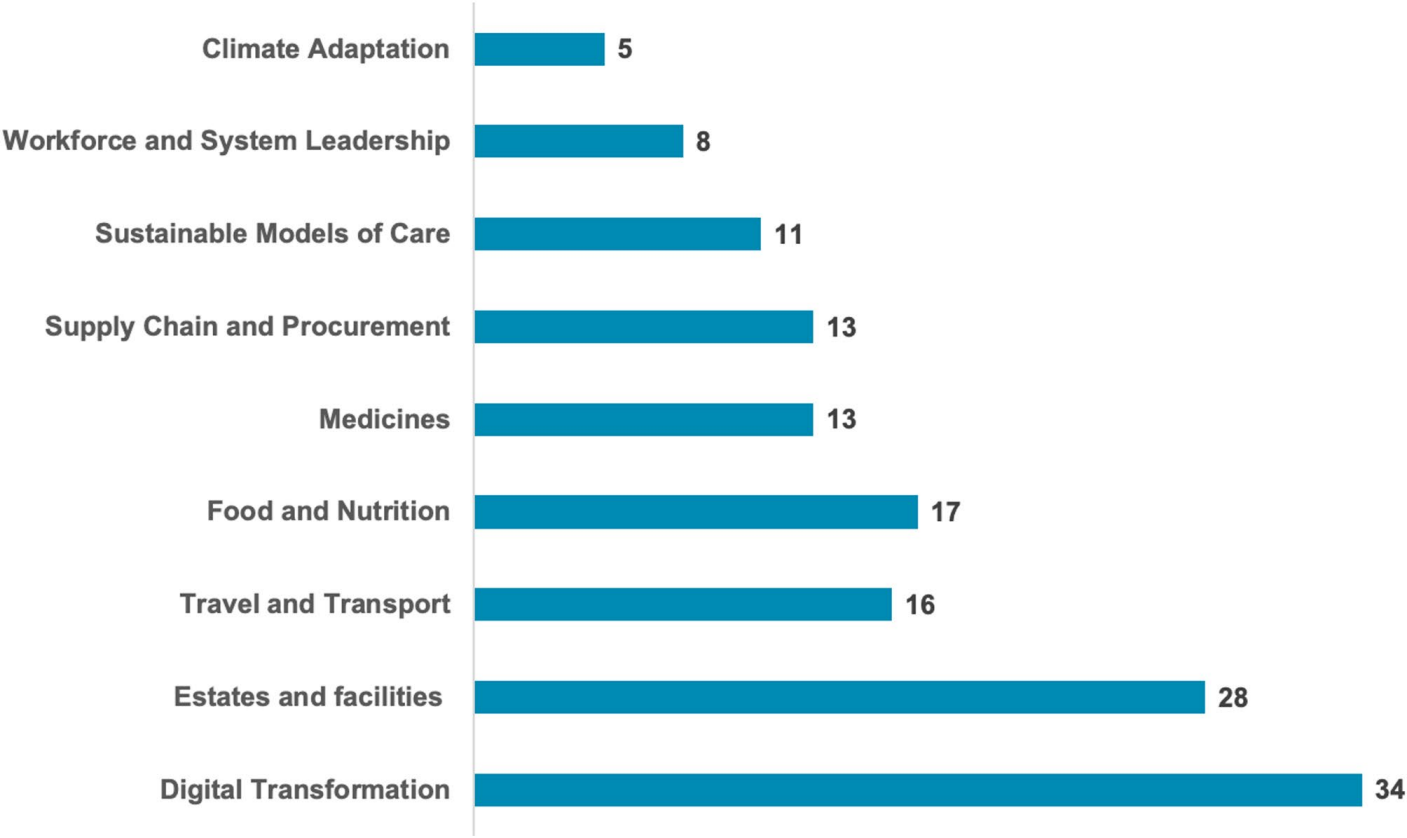
Number of healthcare organizations addressing key areas of environmental sustainability activities

The questionnaire examined how each participating healthcare organization implemented ES across the core areas defined by the Green Plan model. Figure 1 illustrates the prioritization of environmental sustainability initiatives by healthcare organizations in Italy, highlighting the number of organizations working on each area out of a total of 35.

Digital transformation emerges as the most developed area, with 34 organizations actively engaged in its implementation (97%). This reflects a strong focus on expanding the use of telemedicine for remote care and adopting digital systems to reduce reliance on paper records, printing, and postage. These initiatives not only contribute to environmental sustainability but also generate significant cost savings by reducing material usage and administrative expenses. The second most prioritized area is estates and facilities, with 28 organizations reporting active work in this domain (80%). Efforts in this area often focus on improving energy efficiency, reducing energy usage, decarbonizing heating and hot water systems, and adopting waste reduction strategies, including circular economy principles. These initiatives are instrumental in addressing the carbon footprint of healthcare infrastructure while simultaneously reducing operational costs, particularly in energy consumption and waste management. Food and nutrition follow, with 17 organizations involved (49%) in reducing food waste and promoting healthier, locally sourced, and seasonal menus that emphasize plant-based options. This reflects growing recognition of the link between dietary practices and environmental sustainability. Similarly, travel and transport are prioritized by 16 organizations (46%), focusing on increasing active travel and public transport options, as well as investing in low-emission or zero-emission vehicles. These measures also contribute to cost savings by optimizing fleet management and promoting more sustainable modes of transportation. Supply chain and procurement, along with medicines, are addressed by 13 organizations each (37%). In the context of supply chains, efforts aim to reduce the use of clinical and non-clinical single-use plastics, resulting in lower procurement costs and waste management expenses. Initiatives in the area of medicines focus on optimization and waste reduction, responsible disposal, and the consideration of lower-carbon alternatives, all of which can reduce inefficiencies and associated costs. Sustainable models of care are being developed by 11 organizations (31%), emphasizing care delivery closer to home and a preference for lower-carbon interventions when clinically equivalent. These models are not only environmentally beneficial but can also minimize costs related to patient transportation and resource-intensive treatments. Workforce and system leadership, selected by eight organizations (23%), reflects efforts to establish sustainability committees, working groups, and staff training programs on sustainability topics. Although less directly tied to cost savings, these initiatives help embed a culture of sustainability, which can lead to more efficient resource use in the long term. Finally, climate adaptation is the least developed area, with only five organizations (14%) reporting actions such as planning to mitigate the effects of flooding or heatwaves on infrastructure, patients, and staff. While these measures often require significant upfront investment, they have the potential to prevent costly disruptions in the future.

**Fig. 2:**
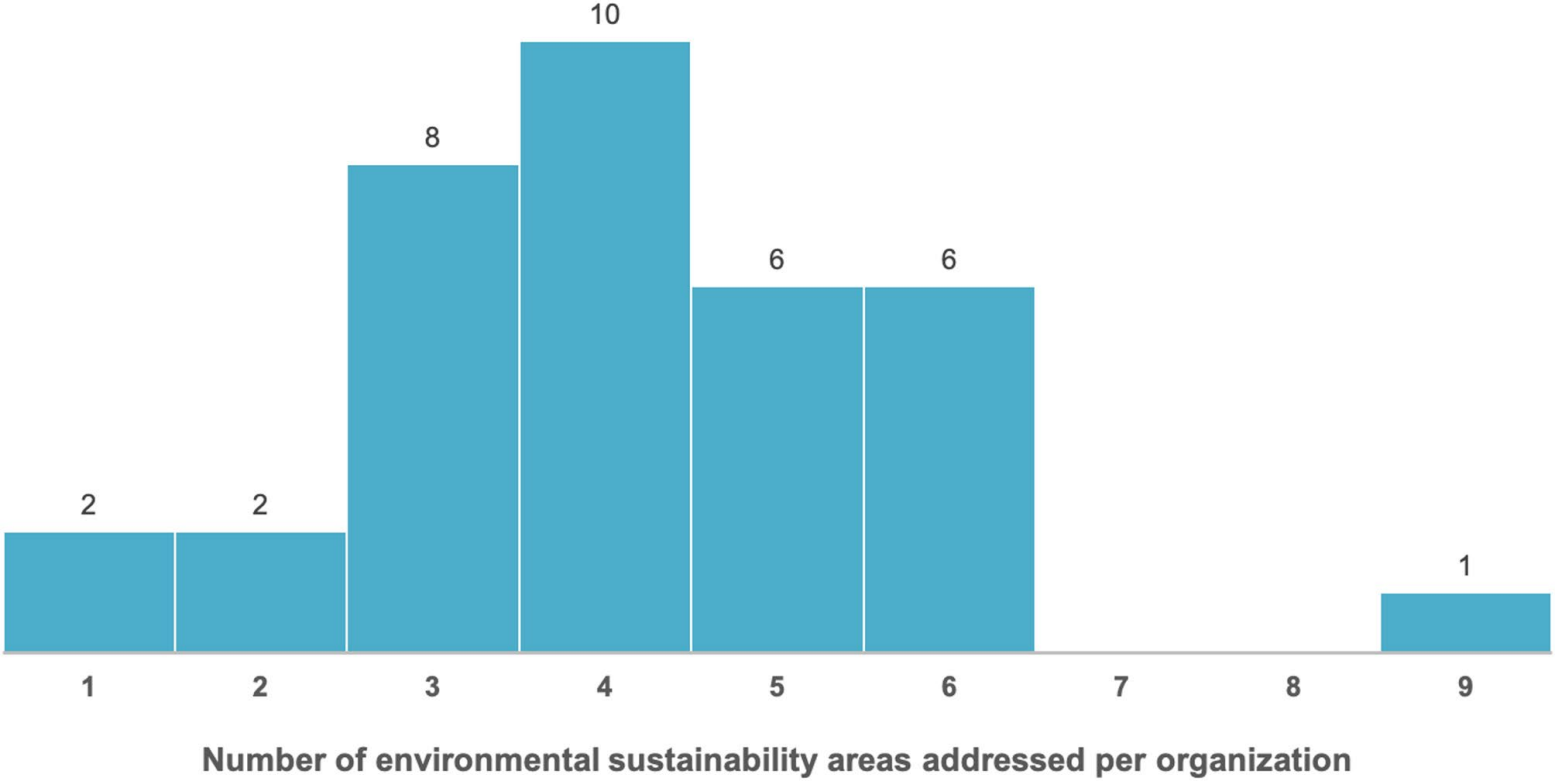
Distribution of the number of environmental sustainability strategy areas addressed by healthcare organizations

Figure 2 illustrates the distribution of the number of environmental sustainability strategy areas addressed by healthcare organizations, highlighting the extent of multi-dimensional engagement.

The image highlights that the majority of healthcare organizations concentrate on approximately four areas when implementing environmental sustainability practices, as reflected by the mean (4.15) and median [4]. This represents a relatively narrow scope compared to the maximum potential engagement across the nine areas proposed in the questionnaire. Notably, 25% of the organizations demonstrate a more systemic approach to environmental sustainability, addressing more than five areas. However, it is important to note that the breadth of their engagement is not always reflected in their strategic documents, as elaborated in the related section.

With regard to the types of actions undertaken for environmental sustainability strategies by healthcare organizations, the results reveal a certain heterogeneity in the concreteness of the activities. The most commonly undertaken activity is the identification of objectives and priorities for making the organization environmentally sustainable, reported by 22 organizations (62.9%). This suggests that setting strategic goals is perceived as a foundational step in addressing sustainability. The development of monitoring systems, such as quantitative indicators (e.g., CO2 emissions) or qualitative ones (e.g., awareness initiatives), was the second most frequent activity, undertaken by 16 organizations (45.7%). The creation of SMART actions—those that are specific, measurable, achievable, relevant, and time-bound—focused on initial efforts to reduce environmental impacts, and broad engagement with internal and external stakeholders were both reported by 15 organizations (42.9%). On average, each organization has implemented two actions (mean = 2), with a standard deviation of 0.998, indicating some variation in the extent of engagement across organizations.

**Fig. 3:**
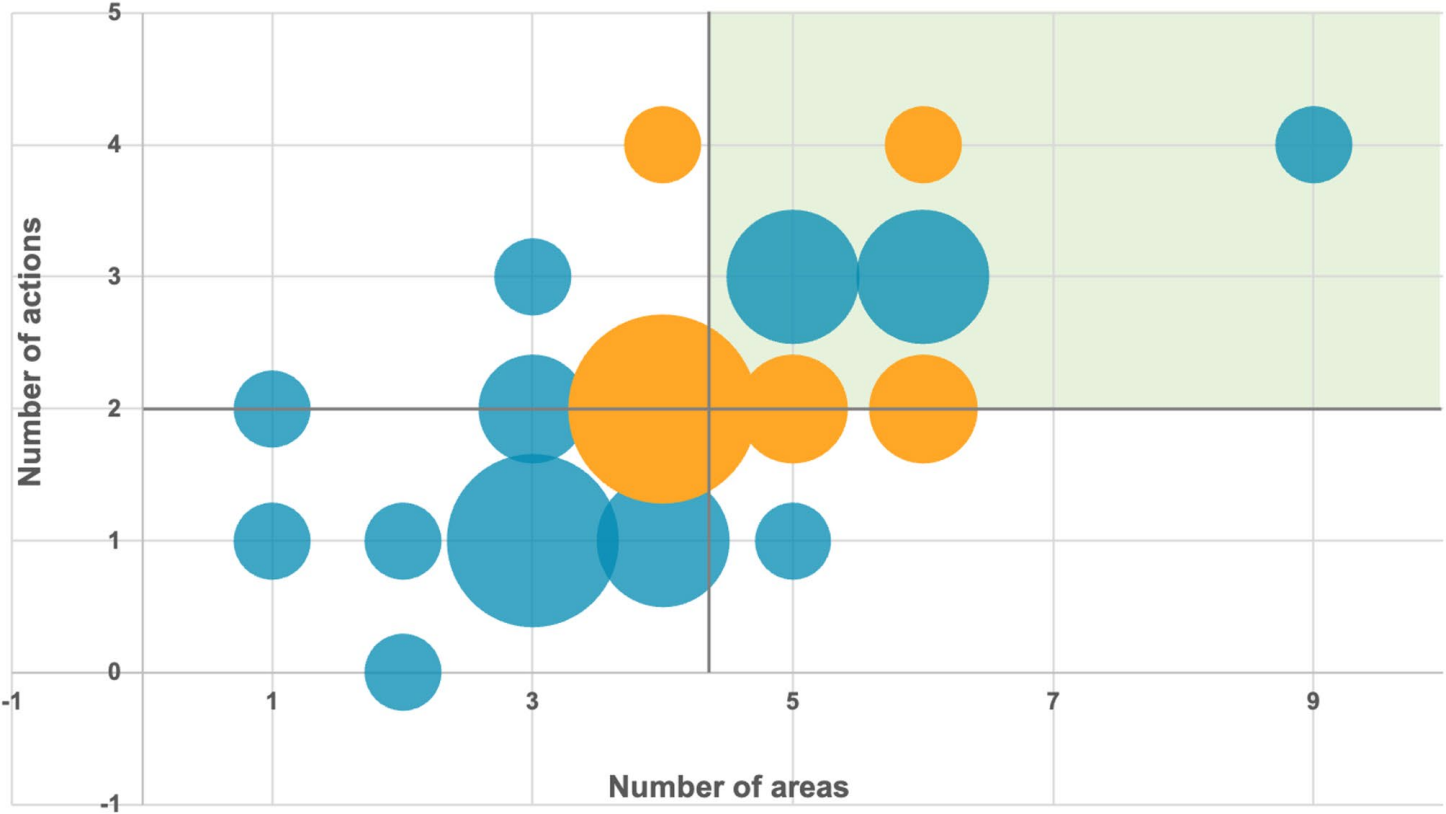
Positioning of healthcare organizations based on the breadth and depth of environmental sustainability implementation. The x-axis represents the number of ES areas addressed (ranging from 0 to 9), while the y-axis indicates the number of concrete actions implemented (ranging from 0 to 4). Bubble size corresponds to the number of organizations. The green-shaded area denotes higher levels of both breadth and concreteness in ES engagement

Figure 3 offers an integrated overview of how healthcare organizations engage with ES, based on two dimensions: the breadth of their approach, measured by the number of ES areas addressed (x-axis), and the depth of their engagement, reflected by the number of actions implemented (y-axis). Each bubble represents one or more organizations, with the size of the bubble indicating how many organizations share that specific position. Bubbles including at least one organization with a dedicated sustainability team are shown in orange, while all others are displayed in blue. The green-shaded area highlights organizations exhibiting broader and more structured ES engagement.

The analysis reveals a direct association between the number of areas addressed and the number of actions implemented. Organizations engaging across a broader range of areas also tend to undertake a greater number of concrete actions. A cluster of organizations appears in the middle of the graph, representing those that have addressed a moderate number of areas and undertaken a limited set of concrete actions. These organizations suggest a balanced but relatively restrained approach to environmental sustainability, focusing on a manageable scope without fully embedding or operationalizing their strategies. Notably, a smaller group of organizations is positioned in the upper-right quadrant, indicating both a broad engagement across multiple areas and a higher level of concrete action. Regarding the presence of dedicated sustainability teams, the findings suggest that while the establishment of such structures does not automatically guarantee an advanced level of implementation, it appears to represent a positive initial step. Organizations with dedicated teams tend to display intermediate to high levels of activity: notably, 2 organizations with a dedicated team report the maximum number of actions implemented (4 actions), while others demonstrate good coverage across 4 to 4 ES areas.

### Barriers and drivers in implementing ES activities

The survey findings highlight several key drivers for environmental sustainability in healthcare organizations, which were gathered through an open-ended question. In contrast, the barriers, derived from those emerging in the literature, were systematically evaluated by the participants in terms of importance. A fundamental driver is the recognition of the close link between environmental health and public well-being. Several respondents stressed that sustainable practices are essential not only for reducing the environmental footprint of healthcare services but also for promoting long-term public health benefits. As one participant noted: “*Environmental sustainability is not just about saving energy; it is about reducing the future burden on the healthcare system by promoting healthier environments*”. In this perspective, healthcare organizations are increasingly seen as central actors in advancing public health, with a responsibility to lead by example. Economic factors also emerge as a significant driver. Participants emphasized the potential for cost savings through initiatives such as energy efficiency improvements, waste reduction, and resource optimization. One respondent highlighted: “*Investments in sustainability*,* such as solar panels and LED systems*,* are not only ethically right but also economically strategic*,* significantly reducing operational cost*”. These economic benefits are often coupled with enhanced operational efficiency, as more sustainable practices—such as energy conservation and waste reduction—often streamline processes and reduce wasteful expenditures. This not only improves financial sustainability but also strengthens an organization’s competitive position within the healthcare sector. Furthermore, sustainability is seen as a means of fostering innovation and gaining a competitive advantage. Many respondents pointed out that adopting sustainable practices allows for the development of new healthcare delivery models and technologies, which can enhance service quality and patient care. Hospitals that embrace sustainability are also viewed more favorably by environmentally conscious patients, staff, and investors, helping them to attract talent and build stronger relationships with key stakeholders. In this sense, sustainability is also a driver of reputation and organizational growth. Regulatory compliance is another critical driver for promoting environmental sustainability. Adhering to international and national environmental standards, such as the Kyoto Protocol and the “Do No Significant Harm” (DNSH) principle under the National Recovery and Resilience Plan (NRRP), is crucial for healthcare organizations. A participant remarked: “*Meeting environmental regulations is essential not only to avoid sanctions but to build community trust and credibility.”* Compliance with these regulations ensures that organizations meet their legal obligations while simultaneously improving their public image and gaining trust from the community. Lastly, there is a strong emphasis on corporate social responsibility (CSR), with top management viewing sustainability as an ethical obligation and a strategic investment for future generations. As one respondent succinctly put it: “*Sustainability is not just a project; it is part of our mission to care for people and the planet at the same time.”* This commitment not only enhances the organization’s image but also fosters a sense of pride and engagement among employees and stakeholders. By viewing sustainability as a long-term investment, healthcare organizations recognize that their actions today will have lasting impacts on the environment and public health, helping to ensure a healthier, more sustainable future.

Table 4 presents the perceived relevance of each barrier identified by participants in relation to the implementation of environmental sustainability strategies (ESS) within their respective organizations.

**Table 4:** Hindering factors to the adoption of environmental sustainability activities in healthcare organizations

Hindering factors	Average score	Min	Max	Std Dev
The collection of environmental data requires time and effort	6.92	2.00	10.00	1.84
Limited data availability/lack of an organizational monitoring system.	6.66	2.00	10.00	1.99
Presence of higher-priority issues to address.	6.42	2.00	10.00	2.00
Absence of a political mandate on this issue.	6.39	2.00	10.00	2.31
Added value not fully perceived by organization members (doctors, nurses, support staff, auxiliary staff, administrative personnel, etc.).	6.16	2.00	10.00	2.40
Limited familiarity with strategies related to environmental sustainability.	6.05	2.00	10.00	2.08
Added value not perceived by patients.	5.61	2.00	10.00	2.11

This table presents the barriers to the implementation of environmental sustainability activities in healthcare organizations. Each factor is evaluated on a scale from 1 to 10, with the average score reflecting the perceived importance of each barrier, alongside the minimum, maximum, and standard deviation values

The analysis of perceived barriers to the implementation of ES activities reveals a relatively balanced distribution of scores, indicating that healthcare organizations face multiple, interrelated challenges rather than a single dominant obstacle. Although the collection of environmental data received the highest average score (6.92), followed closely by the lack of an organizational monitoring system (6.66), the differences across all barriers are moderate, with average scores ranging from 5.61 to 6.92 on a 10-point scale. This narrow range suggests that respondents perceive these barriers as similarly relevant, reflecting the complexity of integrating ES into healthcare settings where technical, organizational, cultural, and contextual factors interplay. For instance, while data-related issues (collection effort and monitoring systems) emerged as slightly more pressing, other factors—such as competing priorities (6.42), absence of political mandates (6.39), and limited awareness or perceived value among staff (6.16)—also represent substantial hindrances. Furthermore, the standard deviation values, generally above 2.0, highlight a degree of variability in perceptions across organizations, likely influenced by differences in internal capacities, governance structures, and levels of familiarity with sustainability practices.

## Discussion

### Main findings

This study provides a comprehensive overview of the level of awareness, degree of implementation, and extent of operationalization of ES activities within Italian healthcare organizations, offering insights that both confirm and expand upon existing literature. Interpreted through the lens of Rogers’ innovation-decision model—comprising the stages of persuasion, decision, and adoption—the findings reveal several noteworthy patterns. In particular, there is a high level of awareness among healthcare leaders (97%), corresponding to the persuasion stage, which aligns with prior research emphasizing the increasing recognition of sustainability’s importance within healthcare management [12, 21]. However, a more unexpected finding concerns the decision stage. The formal incorporation of ES principles into strategic documents appears to be relatively limited, with only 41% of organizations reporting such formalization. This is especially striking given that 90% of participants indicated that they had already undertaken at least some actions related to environmental sustainability, suggesting progression into the adoption phase even in the absence of formal strategic endorsement. While the literature frequently highlights the persistent gap between institutional awareness and formal commitment in public administration [23, 46], the present findings suggest an inverse phenomenon: despite the traditionally formalistic nature of public sector organizations, many healthcare institutions have initiated sustainability actions without prior strategic formalization. Furthermore, the strategic documents provided by respondents generally address environmental sustainability in broad, high-level terms rather than establishing specific operational frameworks to guide strategic choices and priorities [28, 47]. This finding invites reflection on the actual role of formalization: to what extent is it truly necessary to drive sustainability efforts forward, and under which conditions might informal or emergent practices suffice to promote meaningful change?

A noteworthy finding emerging from the analysis is the positive association between the number of ES areas addressed and the number of actions implemented. Organizations that engage across a broader range of ES areas also tend to undertake a greater number of concrete initiatives. This pattern challenges the common assumption that focusing efforts on fewer areas would naturally lead to deeper or more effective implementation. Instead, broader strategic engagement appears to be linked to stronger operational outcomes—potentially supported by institutional commitment and the involvement of multidisciplinary teams. Leadership commitment is consistently identified in the literature as a key enabler of sustainability transitions in complex organizations [14, 37, 38, 48]. When top management actively prioritizes environmental sustainability, organizations are more likely to mobilize the necessary resources and align internal processes with sustainability objectives. In line with this, the presence of dedicated sustainability teams—while not in itself a guarantee of advanced implementation—emerged as a potentially enabling factor. The findings suggest that establishing formal governance structures can be beneficial, but must be complemented by leadership engagement, sufficient resourcing, and mechanisms for organizational learning to translate strategic intentions into tangible results. Consistent with findings from other national and international contexts [10, 11, 13, 14, 20, 21], the actions reported by participating organizations tend to concentrate in areas with immediate, measurable outcomes. These include digital transformation (reported by 97% of respondents) and estates and facilities management (80%), with specific initiatives focused on telemedicine, digital infrastructure, energy efficiency, and waste reduction. These priorities appear to balance environmental benefits with economic rationales, illustrating how financial drivers—highlighted in previous studies [23, 49]—can reinforce organizational engagement. As noted, initiatives such as installing solar panels, adopting LED lighting, and improving waste management not only reduce environmental impact but also yield cost savings and improve operational performance. The issue of priority setting is crucial in explaining these trends. In the post-pandemic period, concerns related to financial stability and care delivery capacity appear to dominate strategic agendas. As a result, environmental objectives may be deprioritized in favor of more immediate clinical or operational demands. This tendency is reflected in the perceived importance of “higher-priority issues” as a barrier to ES implementation. Although the link between environmental and public health, including the broader planetary health agenda, is increasingly recognized [1, 3, 12, 23, 50–52] and was identified by respondents as a relevant motivating factor, this awareness has yet to translate into institutional prioritization. Despite a conceptual understanding of the interdependence between ecosystem health and population well-being, ES continues to face challenges in gaining formal recognition in health policy and strategic planning [24, 28]. The concentration of action in certain domains is also likely influenced by external drivers. For instance, digital transformation is a core component of Italy’s National Recovery and Resilience Plan (PNRR), while energy-related initiatives have been promoted at the regional level, particularly in Tuscany, partly in response to rising energy costs. By contrast, more systemic ES domains—such as climate change adaptation (14%) and sustainable models of care (31%)—remain underdeveloped, consistent with the literature’s observation that sustainability efforts often emphasize short-term efficiency gains over long-term structural change [10, 22]. This lack of systemic engagement is further compounded by the absence of a strong political mandate, as widely documented in prior studies [12, 23, 46, 53]. Without clear institutional directives or supportive policy frameworks, sustainability initiatives may remain peripheral, lacking the visibility and support required for large-scale implementation. Conversely, national commitments—such as the NHS’s target of achieving net-zero emissions [44]—demonstrate the transformative potential of policy leadership in positioning health systems as active contributors to climate and public health goals. Finally, the analysis of perceived barriers to ES implementation reveals that healthcare organizations face a complex array of interrelated challenges, rather than a single dominant constraint. The relatively even distribution of perceived importance across different barriers suggests that no single issue can be addressed in isolation. Nevertheless, the time and effort required to collect environmental data, along with the absence of internal monitoring systems, were perceived as slightly more critical. These findings point to persistent difficulties in measuring, managing, and institutionalizing environmental performance across healthcare organizations.

### Strengths and limitations

The main strength of this study lies in its capacity to provide an overview of how Italian healthcare organizations are engaging with environmental sustainability (ES) in the absence of a strong national directive. It contributes original insights by highlighting an unexpected discrepancy between formal commitment and operational action—surprisingly in favour of the latter. Moreover, the study reveals a positive correlation between the breadth of strategic engagement across multiple ES domains and the depth of action implemented, suggesting that wider involvement may support more concrete operationalization. Nonetheless, several limitations warrant consideration. The findings are derived from survey responses, reflecting the subjective perceptions of participants. This introduces potential biases influenced by individual roles, priorities, or understanding of the organization’s sustainability efforts. While valuable, these insights may not fully capture the objective reality of ES implementation. Moreover, the response rate of 25% limits the generalizability of the findings, as it may reflect a lack of engagement or varying levels of interest in sustainability across the broader healthcare sector. Despite these challenges, the study confirms and contextualizes barriers widely recognized in the literature, such as resource constraints, limited monitoring systems, and competing priorities. However, it adds new evidence specific to the Italian healthcare system, demonstrating how the absence of a centralized policy exacerbates these issues.

## Conclusions

In conclusion, this study sheds light on how Italian healthcare organizations are navigating the challenge of environmental sustainability in the absence of centralized policy guidance. Far from being passive actors awaiting regulatory direction, many organizations are demonstrating initiative, experimenting with concrete actions, and embedding sustainability into selected areas of practice. This signals the emergence of a bottom-up dynamic in which operational pressures, ethical commitment, and local leadership are beginning to compensate for the lack of national coordination. What clearly emerges is that organizations do not necessarily follow a linear, formalized pathway to ES. Instead, the data reveal a more fluid, pragmatic process in which action may precede strategy, and implementation may occur even without formal codification in official plans. This adaptive behaviour, while promising, also raises concerns about consistency, scalability, and long-term integration. In the absence of institutional frameworks or shared benchmarks, sustainability risks remaining fragmented and highly variable across contexts.

The relationship observed between the number of areas addressed and the number of actions implemented offers a compelling managerial insight: adopting a broad lens does not dilute impact—it strengthens it. Organizations that frame sustainability across multiple domains tend to act more decisively, suggesting that a systemic approach can foster momentum and resource alignment. From a leadership perspective, this highlights the importance of creating cross-cutting sustainability strategies, supported by multidisciplinary teams and embedded within governance structures.

On the policy front, the findings call for a shift from fragmented local experimentation to coordinated national vision. The absence of a national policy framework not only generates disparities but also limits learning across institutions. A national strategy—paired with enabling tools such as standardized indicators, reporting mechanisms, and financial incentives—could accelerate adoption while respecting local autonomy.

For future research, we propose an in-depth exploration of the organizations positioned at the extremes of our implementation mapping. These cases—those that combine high breadth and high concreteness—represent laboratories of innovation and potential models for the system. Case study research should examine the enabling conditions behind their success: What organizational cultures, leadership styles, and governance models drive advanced ES engagement? Conversely, understanding the inertia affecting low-performing organizations could inform targeted interventions and capacity-building strategies.

In sum, this study underscores the fact that environmental sustainability in healthcare is not merely a technical or regulatory issue—it is a matter of strategic vision, organizational learning, and cultural transformation. Supporting this transformation requires both empirical inquiry and decisive institutional action.

## Supplementary Information

Supplementary Material 1.

## Data Availability

The data relevant to this study are included within the manuscript.

## Abbreviations

SSN: Servizio Sanitario Nazionale (Italian National Health Service)
ES: Environmental Sustainability
LHAs: Local Health Authorities
IRCCS: Scientific Institutes for Research, Hospitalization, and Healthcare
NRRP: National Recovery and Resilience Plan
CAM: Minimum Environmental Criteria (Criteri Ambientali Minimi)
PIAO: Integrated Plan of Activities and Organization (Piano Integrato di Attività e Organizzazione)
PNRR: National Recovery and Resilience Plan (Piano Nazionale di Ripresa e Resilienza)
STREPIN: Strategy for the Energy Refurbishment of Buildings
PNIEC: Integrated National Energy and Climate Plan (Piano Nazionale Integrato per l’Energia e il Clima)
DNSH: Do No Significant Harm principle
