# Publisher Correction: Chasing environmental sustainability in healthcare organizations: insights from the Italian experience

**DOI:** 10.1186/s12913-025-13357-6

**Published:** 2025-08-20

**Authors:** Michela Bobini, Americo Cicchetti

**Affiliations:** 1 CeRGAS, Health and Social Care Management Research Center, SDA Bocconi School of Management, Milan, Italy; 2 Graduate School of Health Economics and Management, ALTEMS, Catholic University of the Sacred Heart, Rome, Italy; 3 Department of Economic and Business Management Sciences, Catholic University of the Sacred Heart, Rome, Italy

**Correction: BMC Health Serv Res 25, 978 (2025)**

**https://doi.org/10.1186/s12913-025-13158-x**

In the PDF of this article, Figs. 1, 2 and 3 were captured incorrectly due to a typesetting mistake. The incorrect and correct version of the figures are displayed below and the original article has been corrected.

In addition, the affiliations 1 and 2 appeared in incorrect order due to a typesetting mistake. The list of affiliations has been updated in this Correction article and the original article has been corrected.

The publisher apologizes to the authors and readers for the inconvenience caused by these errors.

**Incorrect version of Fig. 1**:


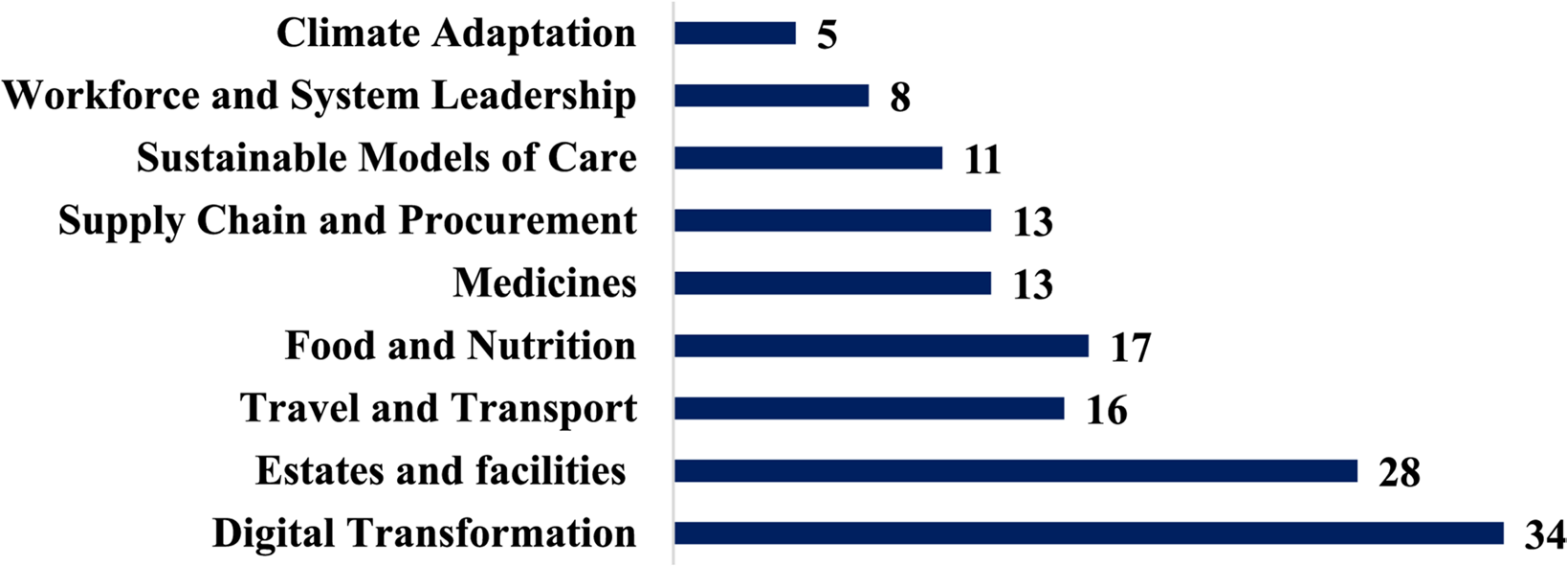


**Correct version of Fig. 1**:

**Fig. 1:**
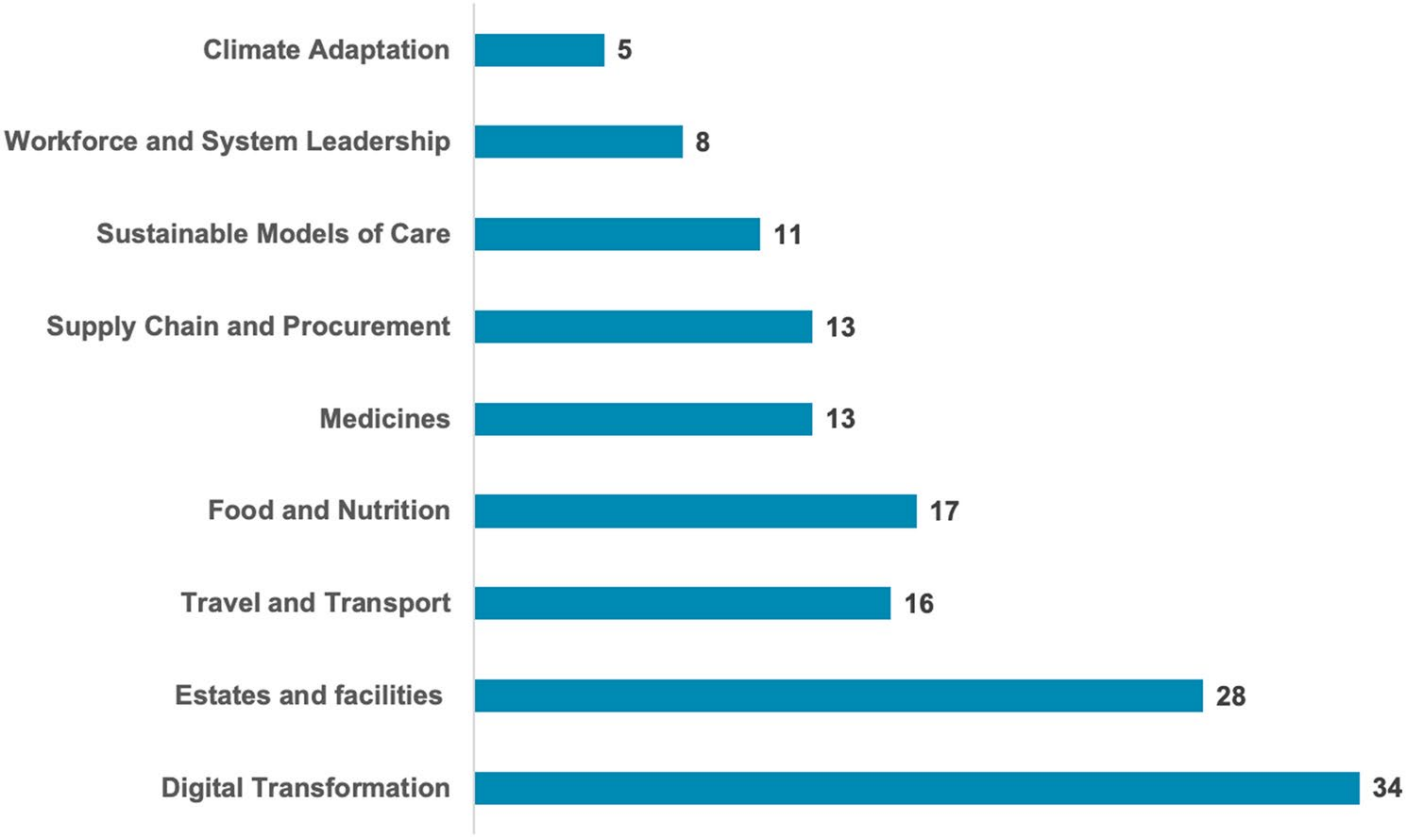
Number of healthcare organizations addressing key areas of environmental sustainability activities

**Incorrect version of Fig. 2**:


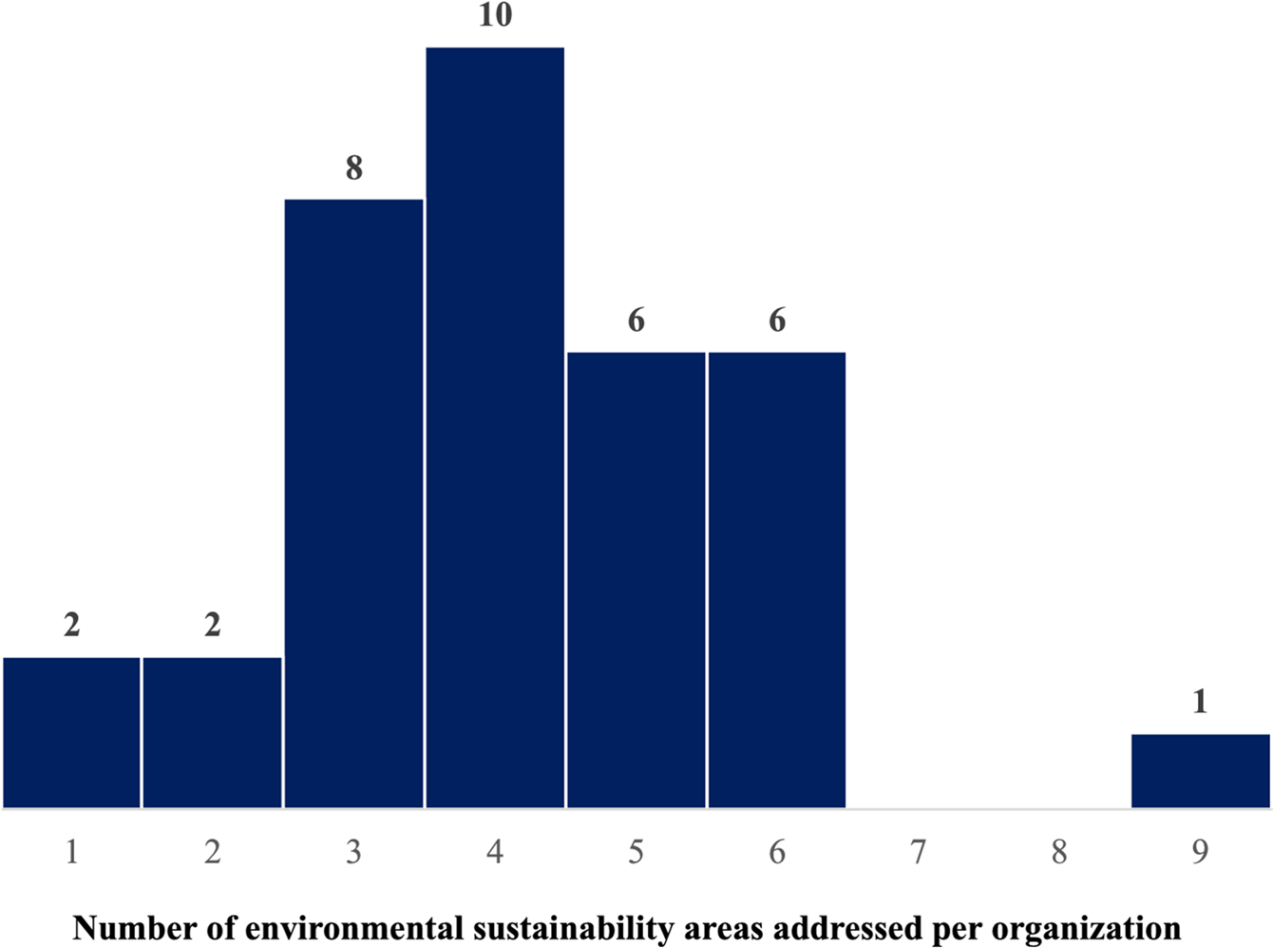


**Correct version of Fig. 2**:

**Fig. 2:**
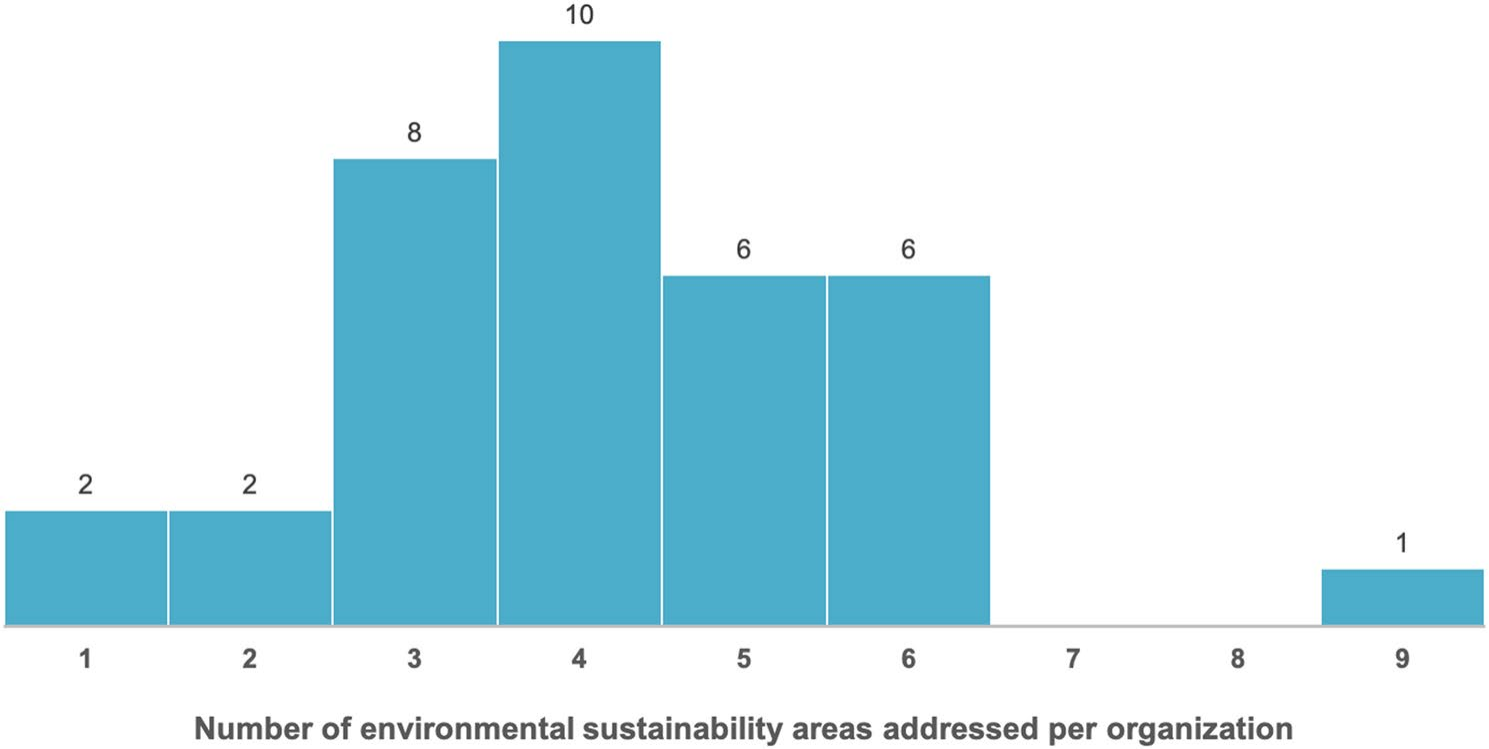
Distribution of the number of environmental sustainability strategy areas addressed by healthcare organizations

**Incorrect version of Fig. 3**:


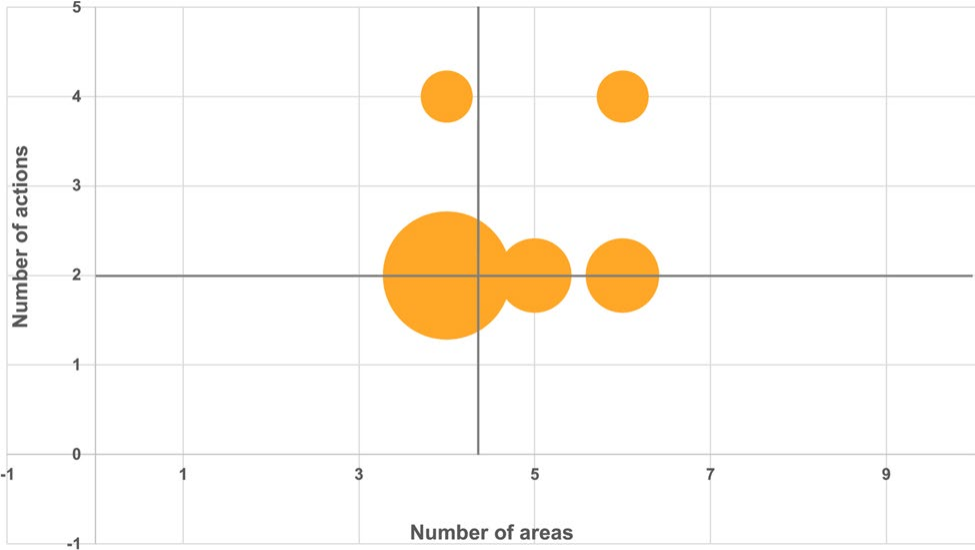


**Correct version of Fig. 3**:

**Fig. 3:**
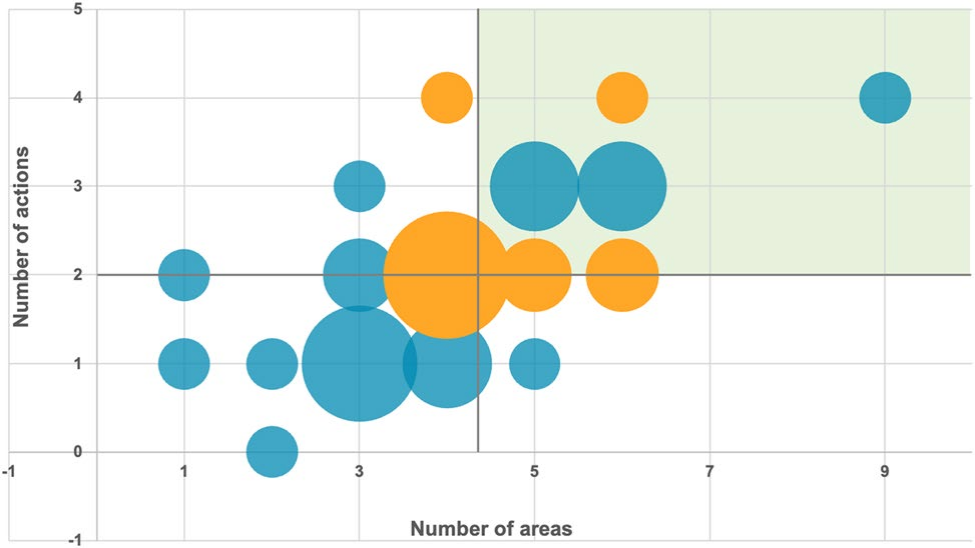
Positioning of healthcare organizations based on the breadth and depth of environmental sustainability implementation. The x-axis represents the number of ES areas addressed (ranging from 0 to 9), while the y-axis indicates the number of concrete actions implemented (ranging from 0 to 4). Bubble size corresponds to the number of organizations. The green-shaded area denotes higher levels of both breadth and concreteness in ES engagement

