# Supplementary material for: Chasing environmental sustainability in healthcare organizations: insights from the Italian experience
Source: BMC Health Serv Res. 2025 Jul 26;25:978. doi: 10.1186/s12913-025-13158-x (PMC12296623; doi:10.1186/s12913-025-13158-x)
Supplement: Supplementary file 1 — Supplementary Material 1. [file 12913_2025_13158_MOESM1_ESM.docx]

*Structure of the survey*

| 1. **Please write the name of the healthcare organization:** 2. **Please indicate the region where your healthcare organization is located:** 3. **Please describe your position inside the organization:** 4. **Please write your email contact for further questions:** 5. **In your region, are there any legislative requirements and/or guidance that specifically promote environmental sustainability in the healthcare sector (and not as part of broader cross-sectoral strategies)?** YES/NO 6. **Please specify which legislative requirements and/or healthcare-specific guidance:** 7. **Is the top management in favor of considering environmental sustainability issues in the management of the healthcare organization?** YES/NO 8. **With reference to the previous question, please specify why the management is in favor or not:** 9. **Has the management formalized the intention to work on environmental sustainability in the healthcare organization?**    - no, ES has not yet been formalized in strategic documents    - yes, ES has been integrated into the strategic documents of the healthcare organization    - yes, there is a document dedicated to the environmental sustainability of the healthcare organization 10. **If yes, please upload the document:** 11. **Have managerial actions been taken to address the issue of environmental sustainability within the healthcare organization?** YES/NO 12. **What are the main drivers and barriers to adopting environmental sustainability practices in your healthcare organization?** YES/NO 13. **Below are some factors that may hinder the adoption of good environmental sustainability practices. Please rate the relevance of each on a scale from 1 to 10.** (1 = not relevant at all, 10 = extremely relevant)     - There are other, more pressing priorities to address     - Lack of data/absence of an internal monitoring system     - Collecting environmental data can be time- and labor-intensive     - Absence of a political mandate on this issue     - Limited familiarity with environmental sustainability strategies     - The added value is not fully perceived by the organization’s members (e.g., doctors, nurses, support staff, admin, etc.)     - The added value is not perceived by patients 14. **Please select one or more activities that have been undertaken in the field of environmental sustainability in your healthcare organization:**     - identification of objectives and priorities to address ES in the healthcare organization;     - development of systems and processes to measure and report on progress against plans and commitments;     - development of SMART (specific, measurable, achievable, relevant and time-bound) actions focused on early efforts to reduce environmental impacts;     - engagement widely with internal stakeholders and key partner organizations to inform sustainability priorities and identify areas for productive collaboration;     - Other, please specify: 15. **Below are some areas for implementing environmental sustainability actions in healthcare organizations (as identified by the UK NHS model). Please select one or more areas in which you have worked on environmental sustainability:**     - Workforce and system leadership (e.g. sustainability committees and working groups, online sustainability training and pledge platforms for staff)     - Sustainable model of care (e.g. provision of care closer to home, default preferences for lower-carbon interventions where clinically equivalent)     - Digital transformation (e.g. expanding use of telemedicine for remote care, digital systems to reduce paper records, printing, and postage)     - Travel and transport (e.g. increasing levels of active travel and public transport, investing in ultra-low emission and zero-emission vehicles)     - Estates and facilities (e.g. improving energy efficiency and reducing energy usage, decarbonizing heating and hot water systems, waste reduction and circular economy)     - Medicines (e.g. medicines optimization and waste reduction, responsible capture or disposal of waste medicines or considering lower carbon alternative medicines)     - Supply Chain and Procurement (e.g. reducing use of clinical and non-clinical single-use plastic items)     - Food and Nutrition (e.g. reducing overall food waste, providing healthier, locally sourced, and seasonal menus high in fruits and vegetables)     - Adaption (e.g. plans to mitigate effects of flooding or heatwaves on infrastructure, patients, and staff)     - Other, please specify: 16. **With regard to the governance of environmental sustainability, have any actions been taken at the organizational level?**     - no     - yes, by assigning the responsibility to a person or team already holding a role within the healthcare organization     - yes, by appointing a sustainability manager     - yes, by hiring an energy manager     - yes, by creating a dedicated team for environmental sustainability     - Other, please specify: 17. **What kind of backgrounds have those involved in the implementation of ES in the healthcare organization?**     - Economic     - Engineering     - Law     - Medicine     - Pharmacy     - Chemistry     - Other (please specify which one) 18. **Please use this space for additional comments:** |
| --- |

*Table. Regional distribution of healthcare organizations contacted for the survey, number of responses, and corresponding response rates*

| **Region** | ****Healthcare Organizations Contacted to Complete the Survey**** | ****Responses**** | ****Response Rate**** |
| --- | --- | --- | --- |
| Abruzzo | 4 | 2 | 50% |
| Basilicata | 3 | 0 | - |
| Calabria | 6 | 0 | - |
| Campania | 17 | 1 | 6% |
| Emilia-Romagna | 15 | 4 | 27% |
| Friuli-Venezia Giulia | 4 | 2 | 50% |
| Lazio | 17 | 3 | 18% |
| Liguria | 8 | 1 | 13% |
| Lombardy | 20 | 10 | 50% |
| Marche | 5 | 1 | 20% |
| Molise | 2 | 0 | - |
| PA Bolzano | 1 | 1 | 100% |
| PA Trento | 1 | 0 | - |
| Piemonte | 13 | 1 | 8% |
| Puglia | 10 | 0 | - |
| Sardegna | 7 | 1 | 14.3% |
| Sicilia | 13 | 2 | 15.4% |
| Toscana | 12 | 1 | 8% |
| Umbria | 3 | 0 | - |
| Valle d'Aosta | 1 | 0 | - |
| Veneto | 9 | 9 | 100% |
| ****Italy**** | **171** | **39** | **23%** |

*Note: The sample includes* ***162 public healthcare organizations*** *affiliated with FIASO, after excluding non-service delivery entities (e.g., agencies and coordinating bodies). Additionally,* ***9 healthcare organizations*** *(8 in Veneto and 1 in Lazio) were contacted directly by the authors based on existing professional networks.*
